# Tuning charge carrier transport and optical birefringence in liquid-crystalline thin films: A new design space for organic light-emitting diodes

**DOI:** 10.1038/s41598-018-19157-9

**Published:** 2018-01-15

**Authors:** Chang-Min Keum, Shiyi Liu, Akram Al-Shadeedi, Vikash Kaphle, Michiel Koen Callens, Lu Han, Kristiaan Neyts, Hongping Zhao, Malte C. Gather, Scott D. Bunge, Robert J. Twieg, Antal Jakli, Björn Lüssem

**Affiliations:** 1 Department of Physics, Kent State University, Kent, OH 44242 USA; 2 Department of Electronics and Information Systems, Ghent University, Ghent, B-9000 Belgium; 3 Department of Electrical Engineering and Computer Science, Case Western Reserve University, Cleveland, OH 44106 USA; 4 Organic Semiconductor Centre, SUPA, School of Physics and Astronomy, University of St Andrews, St Andrews, KY16 9SS United Kingdom; 5 Department of Chemistry and Biochemistry, Kent State University, Kent, OH 44242 USA; 6 Liquid Crystal Institute, Kent State University, Kent, OH 44242 USA; 7 Department of physics, University of Baghdad, Al-Jadriya, Baghdad, 10071 Iraq

## Abstract

Liquid-crystalline organic semiconductors exhibit unique properties that make them highly interesting for organic optoelectronic applications. Their optical and electrical anisotropies and the possibility to control the alignment of the liquid-crystalline semiconductor allow not only to optimize charge carrier transport, but to tune the optical property of organic thin-film devices as well. In this study, the molecular orientation in a liquid-crystalline semiconductor film is tuned by a novel blading process as well as by different annealing protocols. The altered alignment is verified by cross-polarized optical microscopy and spectroscopic ellipsometry. It is shown that a change in alignment of the liquid-crystalline semiconductor improves charge transport in single charge carrier devices profoundly. Comparing the current-voltage characteristics of single charge carrier devices with simulations shows an excellent agreement and from this an in-depth understanding of single charge carrier transport in two-terminal devices is obtained. Finally, p-i-n type organic light-emitting diodes (OLEDs) compatible with vacuum processing techniques used in state-of-the-art OLEDs are demonstrated employing liquid-crystalline host matrix in the emission layer.

## Introduction

Controlling the order in thin films of organic semiconductors down to the molecular level has been recognized as a key to improving the performance of organic electronic devices^1–4^. Several techniques to influence crystal structure and molecular alignment have been introduced, in particular for solution-processed organic semiconductors. For wet-chemical processes, the complex interaction of the semiconductor material with the solvent presents an additional means to influence the molecular orientation. For example, it was demonstrated that a simple off-centre spin-coating method enables the formation of highly aligned molecular packing structure of the organic semiconductor^5^; a solution shearing method alters the π–π stacking distance between co-facially stacked molecules and introduces lattice strain^6^; and chemically tailoring of the organic/metal contact interface was shown to influence the microstructure of solution-cast organic thin films^7^. These processes resulted in molecular structures in organic field-effect transistors with high charge carrier mobility.

However, solution processing is still highly challenging for highly-efficient organic light-emitting diodes (OLEDs) or solar cells consisting of a multitude of functional layers^8,9^. Chemically orthogonal solvents have to be found that are not harmful to pre-deposited films, which significantly restricts the choice of materials and possible number of layers. Although it has been reported recently that OLEDs with even a single organic layer hold promise for high efficiency^10–12^, in general, the device performance is significantly enhanced when utilizing several functioning layers individually, e.g. charge transporting layers and emission layer^13^. More specifically, p-i-n type OLEDs have been extensively studied to take advantage of their possibility of low voltage operation, which is a key factor for high power efficiency leading to reduce the power consumption^14^. Therefore, the challenges for solution processing have led to a slight preference for vacuum deposited OLEDs, where some highly efficient devices consist of up to 10 layers^15^. On the other hand, however, the possibilities to alter molecular alignment of organic films deposited by thermal evaporation are extremely limited in multi-layer OLED stacks; the few demonstrations reported so far using a brush or a rubbing cloth^16,17^. Even though numerous approaches have been proven to preferentially control the morphology or orientation of vacuum-deposited organic molecules, in most cases physical modifications and/or chemical treatments on the underlying layer are involved, which are generally not applicable to organic semiconductor layers^18–20^.

Liquid-crystalline organic semiconductors have the potential to overcome this challenge and to combine vacuum processing with a high control of the thin film structure. The microstructure of liquid-crystalline organic semiconductors can be altered by manipulating a mesophase using thermal annealing. For example, it was reported that the liquid-crystalline organic semiconductor material, 2,7-dioctyl[1]benzothieno[3,2-b][1]benzothiophene (C8-BTBT), shows a transition between crystalline solid (Cr) and smectic A (SmA) phases during heating and cooling cycles^21^. Thus, the rich phase behaviour of liquid-crystalline semiconductors can be used to control their molecular alignment and e.g. enhance charge transport^22–24^. In addition, liquid-crystalline organic semiconductors show pronounced electrical and optical anisotropies, which provide a wide-range of opportunities to optimize charge carrier transport in organic semiconductors and to tune the light propagation in optoelectronic devices, e.g. OLEDs^25^.

Overall, tuning charge transport and optical birefringence in the functional layers of an OLED has several advantages: Considering that resistive losses inside the intrinsic emission layer is an additional source of luminous efficacy roll-off at high brightness, it is important to increase the electrical conductivity of non-doped layers^9,26^. Hence, being able to tune the charge transport properties of the emission layer can lead to a better charge balance and wider recombination zones, both improving the efficiency of the device. Furthermore, tuning the birefringence in the OLED stack has the potential to shape the emission characteristic of the dipole inside the emission layer and by that to increase the outcoupling efficiency of OLEDs^27^. Similarly, Moon *et al*. reported that the efficiency can be increased when the light is emitted in an optically birefringent medium compared to an isotropic medium, which is successfully verified using experimental data and an optical simulation model^28^.

A number of liquid crystal-based devices have been developed that show promising charge transport^29^, photovoltaic^30^, or light emitting properties^31^. A few authors discussed solution processed OLEDs using light emitting liquid-crystalline materials^32–34^, but a relatively low efficiency was observed, which limits the prospects of these devices. In addition, these studies focus on using emissive liquid-crystalline materials, and the potential of using liquid-crystalline semiconductors as host material in the emission layer for highly efficient optoelectronic devices has not yet been comprehensively investigated.

In this study, we report on tuning the molecular orientation in a vacuum processed liquid-crystalline semiconductor film, C8-BTBT, by a novel blading process as well as by different annealing protocols. Applying the blading process at elevated temperatures, i.e. in a mesogenic phase of C8-BTBT, induces a more tilted alignment from the surface normal. The altered alignment created is verified by cross-polarized optical microscopy and spectroscopic ellipsometry. It is shown that a change in alignment of the liquid-crystalline semiconductor profoundly influences charge transport in hole and electron only devices. The current-voltage characteristics of n-i-n and p-i-p devices are modeled by a space charge limited model as well as a Gaussian disorder model, which show a good agreement with the experimental data. Finally, we demonstrate p-i-n type OLEDs employing C8-BTBT doped with a phosphorescent emitter as an emission layer to explore how the liquid-crystalline semiconductor plays a role as host matrix. The dependence of the emission characteristics and efficiency on the processing conditions is analyzed.

## Results and Discussion

### Alignment Control in C8-BTBT thin-films

**Figure 1:**
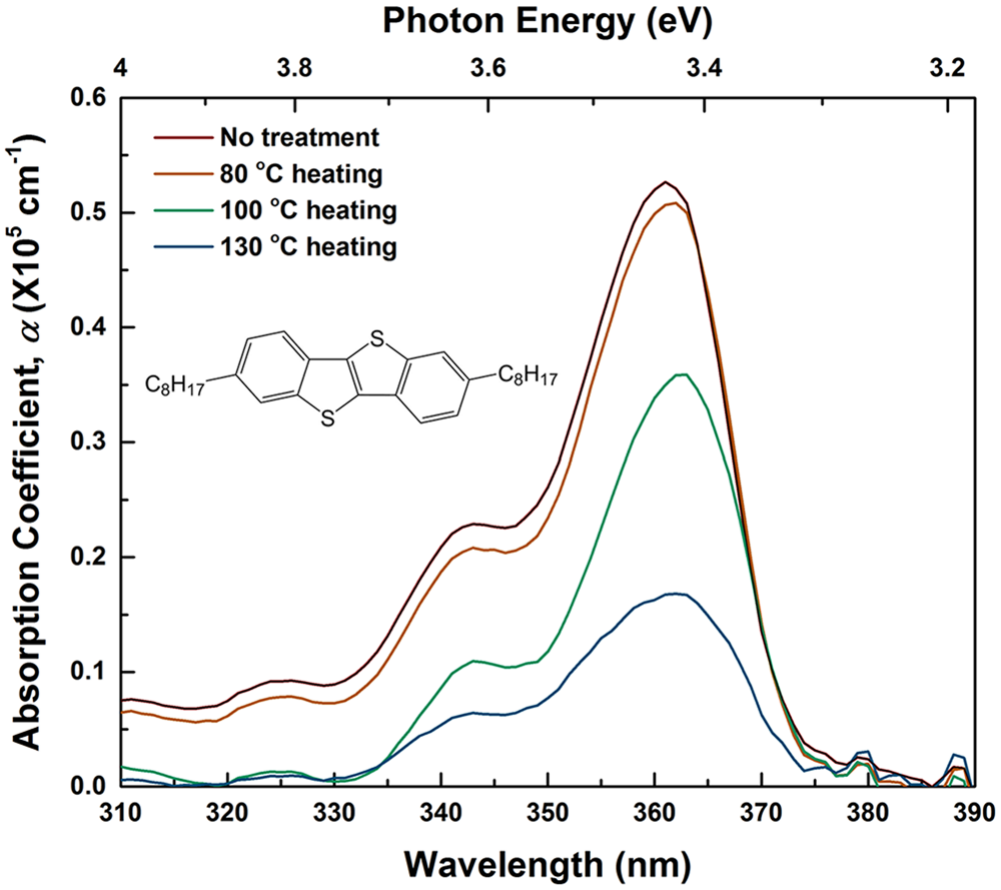
Absorption coefficient *α* of C8-BTBT thin-films (40 nm) annealed at varied temperatures vs. wavelength. Inset: chemical structure of C8-BTBT.

Figure 1 shows optical absorption spectra of neat C8-BTBT thin-films annealed at varied temperatures (see Methods for details of film preparation). The spectra are taken at room temperature (RT) after letting the substrates cool to RT. The absorption coefficient *α* clearly decreases with increasing annealing temperatures. In addition, the peak intensity slightly shifts towards lower energy, i.e. from 3.43 eV to 3.42 eV, if the films are heated above 100 °C. Note that C8-BTBT changes from the Cr to the SmA phase at 110 °C and from SmA to isotropic (I) at 126 °C during the heating cycle. The I–SmA and SmA–Cr transitions occur at 127 °C and 100 °C, respectively, on cooling^21^, but the phase transition temperature in thin-films can differ from the bulk values^35^. Furthermore, it is known that the absorption intensity and peak position are strongly dependent on the incident direction of the light with respect to the orientation of C8-BTBT molecules^36^. Since the non-polarized incident light is kept to be normal to the substrate plane, the variation in the spectrum after heating could be explained by a change in the molecular orientation from the as-prepared initial state.

**Figure 2:**
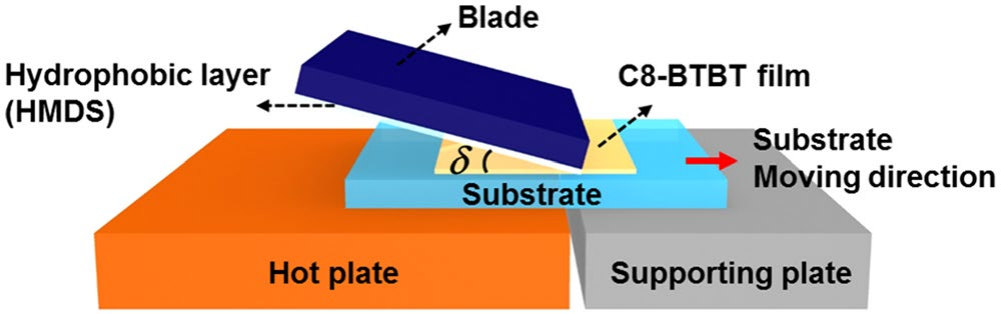
Illustration of the thermal-assisted blading setup. The blade is oriented at a certain angle *δ* with respect to the sample substrate.

To alter the molecular orientation further, we developed a novel blading technique as schematically illustrated in Fig. 2. In the field of organic electronics, blade coating is almost exclusively used for solution processing^6,37–39^. In our study, it is used on vacuum deposited thin-films in their liquid-crystalline phases. A moving stage (not shown in the figure) moves the sample substrate at a constant speed from a hot plate to a supporting plate. While the substrate cools down at the edge of the hotplate, a blade treated with a hydrophobic layer (hexamethyldisilazane (HMDS)) presses onto the softened film, which alters the orientation of the liquid-crystalline C8-BTBT molecules (see Methods for the detailed process).

**Figure 3:**
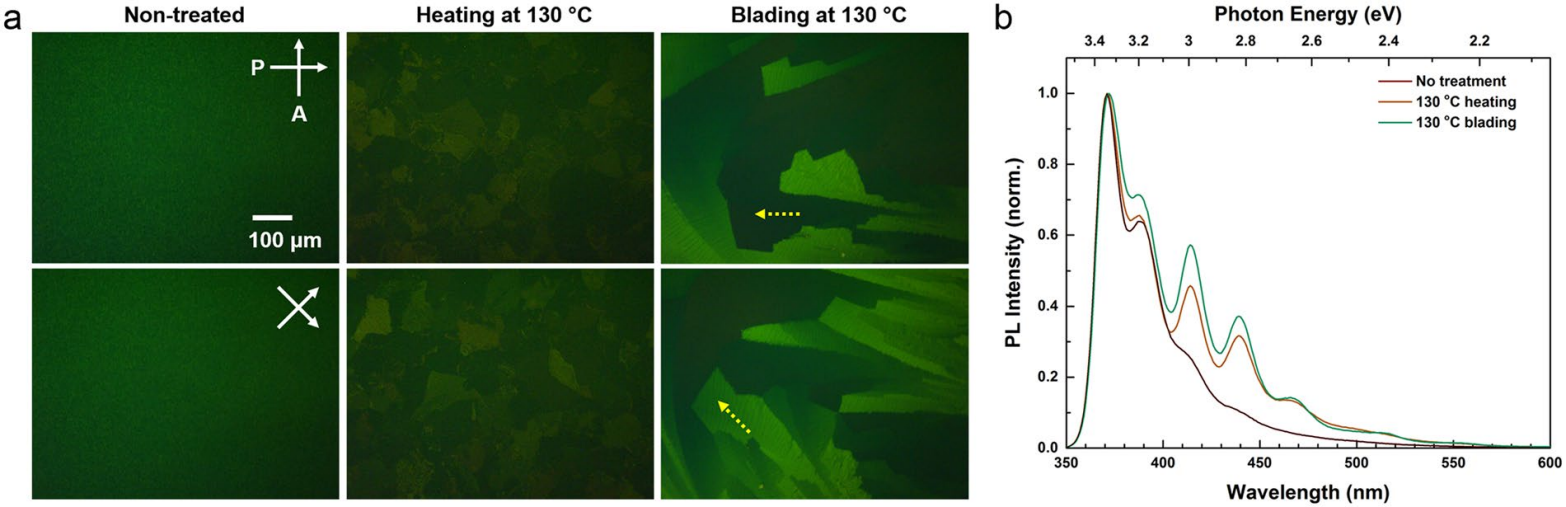
(**a**) Cross-polarized microscopic images of C8-BTBT films (60 nm) without treatment (left) and treated by heating (center) or blading (right) at 130 °C. The lower images are taken after rotating the samples by 45° with respect to the original orientation. The yellow arrows indicate an exemplary molecular domain that exhibits a strong contrast. (**b**) Normalized PL spectra of as-prepared, annealed and thermally assisted bladed C8-BTBT films (40 nm).

Figure 3a shows images of C8-BTBT films treated only by heating or by the blading process, which are taken by a cross-polarized optical microscope in the reflection mode. In case of the as-deposited, non-treated film, an isotropic pattern is observed, which is consistent with previous research that shows the C8-BTBT molecules are homeotropically aligned along the elongated molecular shape (see inset of Fig. 1)^40,41^. By heating to 130 °C, the molecules form random domains that exhibit a slight contrast change seen in the cross-polarized optical microscope image when rotating the samples by 45°. If the films are treated by the blading process, a much stronger contrast is seen (Fig. 3a, right). The yellow arrows indicate one particular domain that is brighter in the lower image. This change in contrast indicates that thermal-assisted blading indeed alters the orientation of C8-BTBT molecules and induces a more birefringent (horizontal) orientation. Furthermore, as seen in Fig. 3b, the photoluminescence (PL) spectra of C8-BTBT films (excitation wavelength, λ_exc_ = 325 nm) change significantly if the films are heated or subjected to the blading process. Annealing and blading leads to sharper peaks in the spectrum and two additional peaks (at 466 nm and 515 nm) emerge.

The appearance of the two additional peaks in the PL spectrum indicate a transition from a less ordered, quenched state of the film generated by vacuum deposition with the substrate held at RT toward a more ordered state in the annealed film. The increase in order will lead to stronger molecular interaction and an increase in the overlap of vibrational wave functions, resulting in the enhancement of emission at corresponding wavelengths^42,43^.

**Figure 4:**
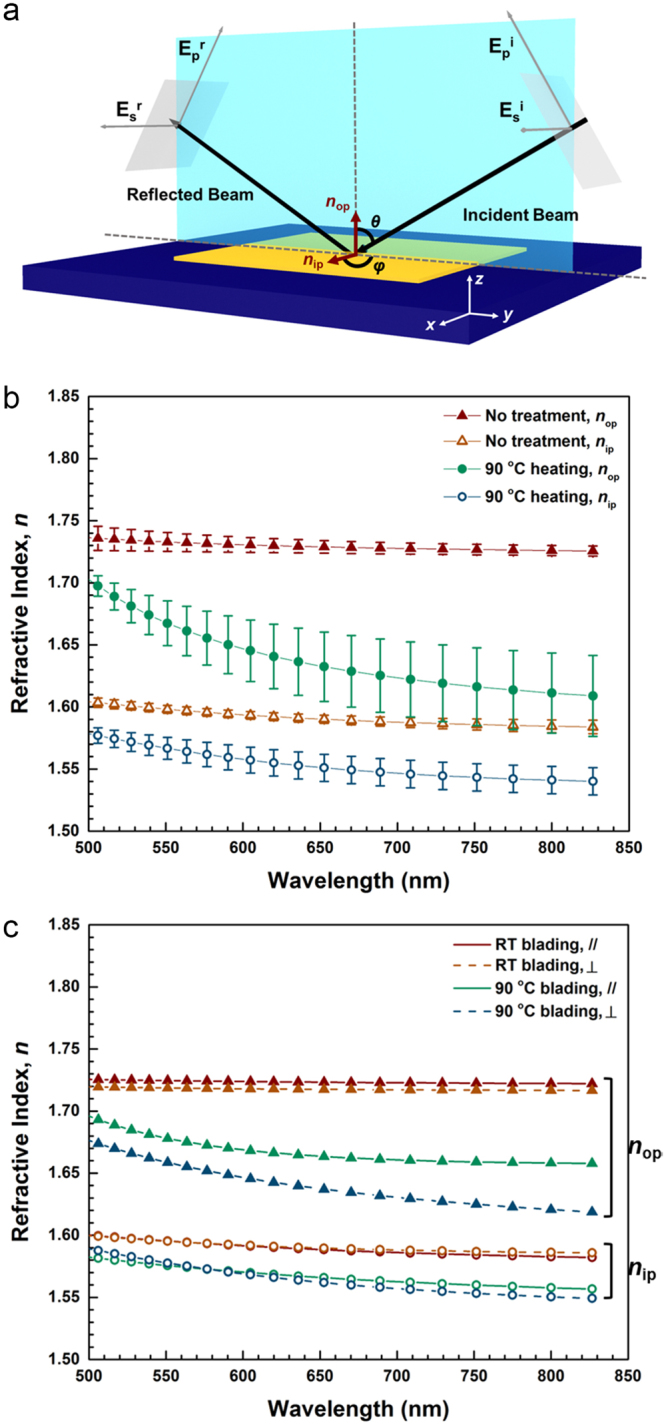
(**a**) Schematic of the spectroscopic ellipsometry setup. The *n*_op_ and *n*_ip_ represent the out-of-plane and in-plane refractive indices of C8-BTBT films, respectively. The angle of the incident beam with respect to the normal direction (*z*-direction) and the azimuthal angle are denoted as *θ* and *φ*. (**b**) Refractive indices of C8-BTBT films without and with heating at 90 °C. The data shown are the average of 4 different measurements ± s.d. (error bar). (**c**) Refractive indices of C8-BTBT films treated by blading at RT and 90 °C. The // and ⊥ symbols indicate the incident beam direction parallel and perpendicular to the blading direction, respectively. The closed triangles represent *n*_op_ and the open circles *n*_ip_. The refractive indices are calculated from the spectroscopic ellipsometry data measured at *θ* = 70° using the modeling implemented in the DeltaPsi2 software platform.

Spectroscopic ellipsometry is used to quantitatively investigate the molecular orientation of C8-BTBT molecules in the film. Figure 4a shows a schematic of the ellipsometry setup. The electric field of the incident and reflected beams is decomposed into a component parallel to the plane of incidence (E_p_) and a perpendicular component (E_s_). The reflection of both components depends on the incident polar and azimuthal angles *θ* and *φ* as well as the shape and the orientation of the molecules in the films, which leads to change in polarization of the reflected beam. If a uniaxial molecule is homeotropically aligned, the polarization of the reflected beam becomes independent of *φ*, leading to identical results even if samples are rotated around the normal direction. However, in the case of a uniaxial molecule tilted from the surface normal, the measured parameters vary significantly with *φ*. Here, the out-of-plane (*n*_op_) and the in-plane (*n*_ip_) refractive indices are defined, respectively, as depicted in Fig. 4a. Note that for a uniaxial material with homeotropic alignment *n*_op_ and *n*_ip_ correspond to the extraordinary (*n*_e_) and the ordinary (*n*_o_) refractive indices, respectively.

The estimated refractive indices of non-treated C8-BTBT films and those heated at 90 °C are plotted in Fig. 4b. In case of the non-treated films, *n*_op_ ~ 1.74 and *n*_ip_ ~ 1.6 at 500 nm with only weak dispersion (wavelength dependence). The measured *n*_op_ = 1.74 is close to the extraordinary refractive indices of typical calamitic liquid crystals confirming that the non-treated, as-deposited C8-BTBT molecules are predominantly aligned vertically along the long optical axis, which verifies the cross-polarized microscopy study shown in Fig. 3a. The birefringence Δ*n* = *n*_e_ − *n*_o_ of the non-treated material is Δ*n*_nt_ ~ 0.14. The effective refractive indices *n*_op_ and *n*_ip_ of the films decrease upon annealing at 90 °C (1.7 and 1.58 at 500 nm, respectively) compared to the non-treated films, and show much greater dispersion. The dispersion can be fitted by the Cauchy formula $n(\lambda )=n(\infty )+a/{\lambda }^{2}+b/{\lambda }^{4}$, which results in *n*_op_(∞) = 1.60, *a*_op_ = −0.00259 (µm^2^), *b*_op_ = 0.00716 (µm^4^) and *n*_ip_(∞) = 1.52, *a*_ip_ = 0.0092 (µm^2^), *b*_ip_ = 0.00116 (µm^4^). The decrease of the extraordinary index can be attributed to the director tilt by an angle *θ*_o_ with respect to the homeotropic alignment, as

1 $${n}_{{\rm{op}}}={n}_{e}^{{\rm{eff}}}=\frac{{n}_{o}{n}_{e}}{\sqrt{{n}_{o}^{2}{\cos }^{2}{\theta }_{o}+{n}_{e}^{2}{\sin }^{2}{\theta }_{o}}}$$

Depending on the wavelength, one can estimate a tilt angle *θ*_o_ varying between 12° and 20° from the measurement results in Fig. 3b.

Figure 4b shows as well that the standard deviation (s.d.) of the refractive indices of the annealed samples is relatively large. The s.d. is obtained from four different measurements, i.e. at two different spots with two different incident beam directions (differing by 90°). This larger s.d. can be explained by a random in-plane orientation of the crystalline domains of the annealed films *φ* (cf. Fig. 3a), which will lead to a large spread in results if the sample is rotated.

In Fig. 4c, bladed films are measured with the incident beam either parallel or perpendicular to the blading direction denoted by // and ⊥ symbols. If this process is done at RT, no significant change in the ordinary or extraordinary refractive indices are observed. However, a remarkable difference (e.g. Δ*n*_op_ = *n*_op_,_//_ − *n*_op_,_⊥_ = 0.03 at 708 nm) is seen when the films are bladed at 90 °C. Treating the films by the blading process (Fig. 4c) leads to a qualitatively similar dispersion of *n*_op_ and *n*_ip_ as in the annealed films, but reduces the spread of the measurements significantly. Hence, blading not only leads to an increase in the tilt angle of the molecules, but induces a preferential in-plane orientation of the tilt *φ* as well. Note that no substantial change of the film thickness by heating and blading is found, which is confirmed by ellipsometry measurements (see Fig. S1 in the Supplementary Information).

### Influence of alignment on vertical charge transport

**Figure 5:**
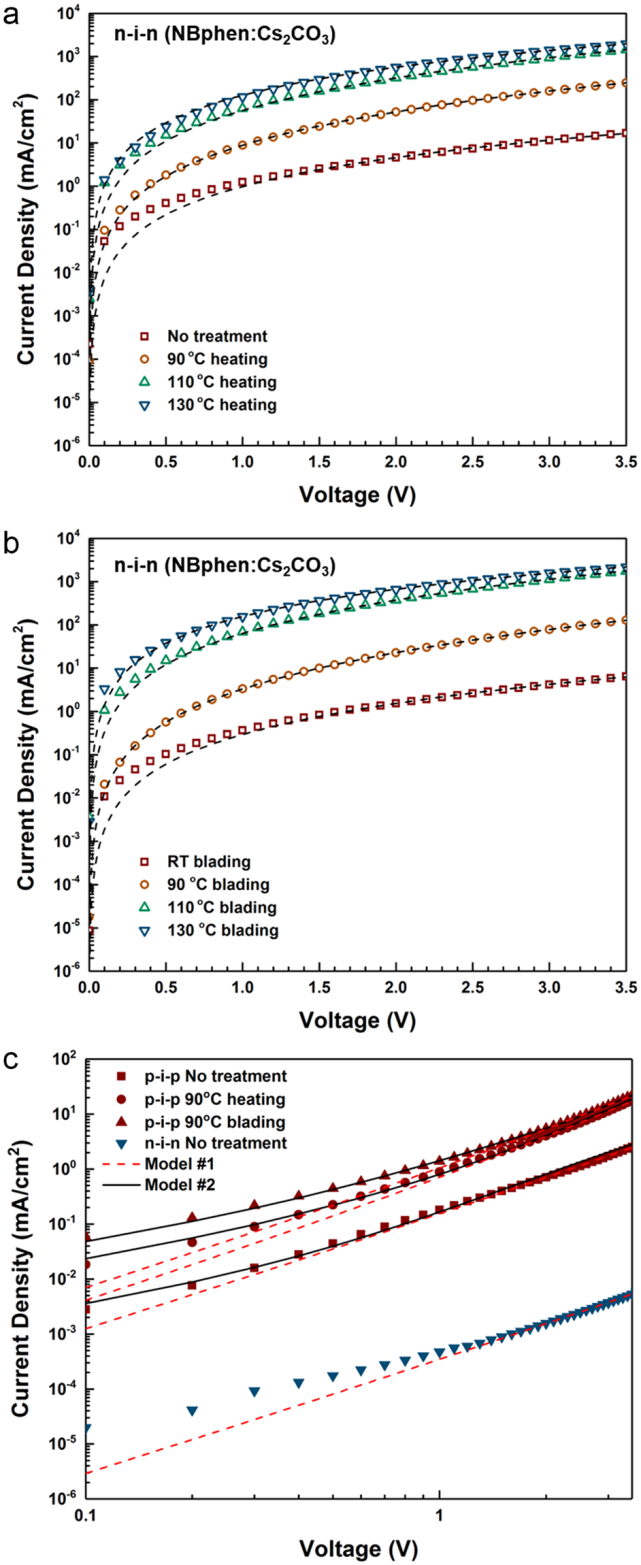
Current density vs. voltage characteristics of n-i (30 nm)-n (NBphen doped with Cs_2_CO_3_) devices treated by (**a**) simple heating and (**b**) our blading technique at varied temperatures. The black dashed lines are the best fit to the data obtained by the modified M-G model. (**c**) Current density of p-i (90 nm)-p (Spiro-TTB doped with F_6_-TCNNQ) and n-i (90 nm)-n (Bphen doped with Cs_2_CO_3_) devices. The red dashed and black solid lines represent the fitting results by the modified M-G model (Model #1) and EGDM (Model #2), respectively.

Single charge carrier devices have been widely studied to understand the hole and electron charge transport properties separately. These devices typically consist of a p(or n)-doped layer, an intrinsic layer, and a p(or n)-doped layer in a sandwiched structure^44^. The doped charge transport layers surrounding the intrinsic layer ensure quasi-Ohmic contacts at the interface to the metallic contacts, which reduces undesirable charge injection losses, and which forces the built-in potential to zero to simplify modelling^45^. Figure 5a,b show the current density characteristics of n-i-n devices treated by simple heating and our thermal-assisted blading technique, respectively, at varied temperatures. We make use of NBphen doped with Cs_2_CO_3_ for n-doped layers to take advantage of their high thermal stability. Here, the voltage is applied to the top electrode while the bottom electrode is grounded. The heating process is performed in a glovebox after the intrinsic C8-BTBT film is deposited on the first doped layer, and the remaining layers are deposited subsequently (see Methods for the detailed protocol). As expected from the results discussed above, the devices treated at a higher heating temperature exhibit a larger current density. This can be attributed to the tilted molecular orientation, which leads to a stronger orbital overlap between adjacent molecules and hence more efficient charge transport in the vertical direction.

Generally, electron and hole mobilities can be extracted from current density–voltage characteristics of single carrier devices using space charge-limited current (SCLC) models. The Mott-Gurney (M-G) relation is widely used to express the current density, yet this model is only valid under the assumptions that (i) the charge carrier mobility is constant and in particular independent of the electric field and the charge carrier density and (ii) any trapping effects are absent^46^. Our devices cannot be satisfactorily modeled by the M-G relation (see the Supplementary Information), and thus, instead, we adopt a modified M-G model taking a field dependency into account for the charge transport modelling^47^.

2 $$J=\frac{9}{8}{\varepsilon }_{r}{\varepsilon }_{0}\frac{{V}^{2}}{{d}_{i}^{3}}{\mu }_{0}\exp (0.89\gamma \sqrt{\frac{V}{{d}_{i}}})$$

where *ε*_r_ is the dielectric constant of the intrinsic layer, *ε*_0_ permittivity, *V* applied voltage, *d*_i_ the intrinsic layer thickness, *μ*_0_ zero-field mobility, and *γ* the characteristic factor for the field-dependence of the Poole-Frenkel model given by $\mu ={\mu }_{0}\exp (\gamma \sqrt{V/{d}_{{\rm{i}}}})$. In Fig. 5a,b, the black dashed lines are best fits to the experimental data, which is obtained by the modified M-G model. The zero-field mobility is found to be dramatically increased from 6.11 × 10^−8^ cm^2^/Vs (non-treated) to 8.79 × 10^−6^ cm^2^/Vs and 1.22 × 10^−5^ cm^2^/Vs by heating alone and heating and blading at 130 °C, respectively. Although this model estimates the overall trend well and describes the date in the high voltage regime accurately, it deviates from the experimental data at low voltages, i.e. for voltages less than 1 V. This might be attributed to the energy barrier at the interface between C8-BTBT and doped layers.

In Fig. 5c, the current density vs. voltage characteristics of as-prepared and treated p-i-p devices are compared to n-i-n devices that consist of Bphen layers doped with Cs_2_CO_3_ which serve as the electron transport layer in the OLEDs studied in the next section. (90 nm thick intrinsic layer). Note that the maximum temperature for the thermal treatment on the C8-BTBT layer in p-i-p devices is limited, due to the instability of the p-dopant, F_6_-TCNNQ, doped into the underlying p-layer at a high temperature (>~100 °C). By annealing and blading, hole transport is improved in the same way as electron transport (cf. Fig. 5a). Lyu *et al*. reported that the energy level of C8-BTBT thin-film shifts depending on the orientation of molecules, i.e. either lying parallel to the plane or standing vertically^48^, which is a phenomenon observed in other anisotropic organic molecules as well^49,50^. Considering that a shift of the energy level occurs in a single direction, i.e. either upward or downward while the gap of the highest occupied molecular orbital (HOMO) and the lowest unoccupied molecular orbital (LUMO) is unchanged and that vertical transport of both holes and electrons is increased by the same treatment, it can be concluded that this is mainly ascribed to a better orbital overlap in vertical direction led by the molecular reorientation even if a possible energy level change is taken into account. Note that it is also found that the current density systematically scales with the thickness of the intrinsic layer for both p-i-p and n-i-n devices (See Fig. S2 in the Supplementary Information). For these p-i-p and n-i-n devices, the modified M-G model provides reliable fitting results, i.e. fitting parameters are in the same order regardless of intrinsic layer thickness, but also shows a discrepancy from the experimental data in a low voltage regime as shown in Fig. 5c.

To quantify the influence of annealing and blading for p-i-p devices more precisely, we employ a simulation model based on the extended Gaussian disorder model (EGDM) developed by Pasveer and Coehoorn *et al*.^51–53^. In this model, the mobility is defined to be not only dependent on the electric field and the temperature but on the charge carrier density as well. For these simulations, a commercial simulation program, ATLAS (Silvaco), which implements the EGDM, is used (see Method and the Supplementary Information for detailed discussion). The energy barriers between adjacent layers are explicitly taken into account. A Poole-Frenkel mobility model is used for the highly doped contact layers and EGDM mobility for the intrinsic layer. Finally, a Gaussian density of states is assumed in the intrinsic layers. As shown in Fig. 5c, the EGDM (Model #2) provides a better fit than the results by the modified M-G model (Model #1). The zero-field mobilities of p-i-p devices as-prepared and treated by annealing and blading at 90 °C are estimated to be 3.5 × 10^−7^ cm^2^/Vs, 8.0 × 10^−7^ cm^2^/Vs, and 1.4 × 10^−6^ cm^2^/Vs, respectively, which are comparable to the values obtained by the modified M-G model (2.82 × 10^−7^ cm^2^/Vs, 7.95 × 10^−7^ cm^2^/Vs, and 1.42 × 10^−6^ cm^2^/Vs).

### Liquid-crystalline OLEDs

**Figure 6:**
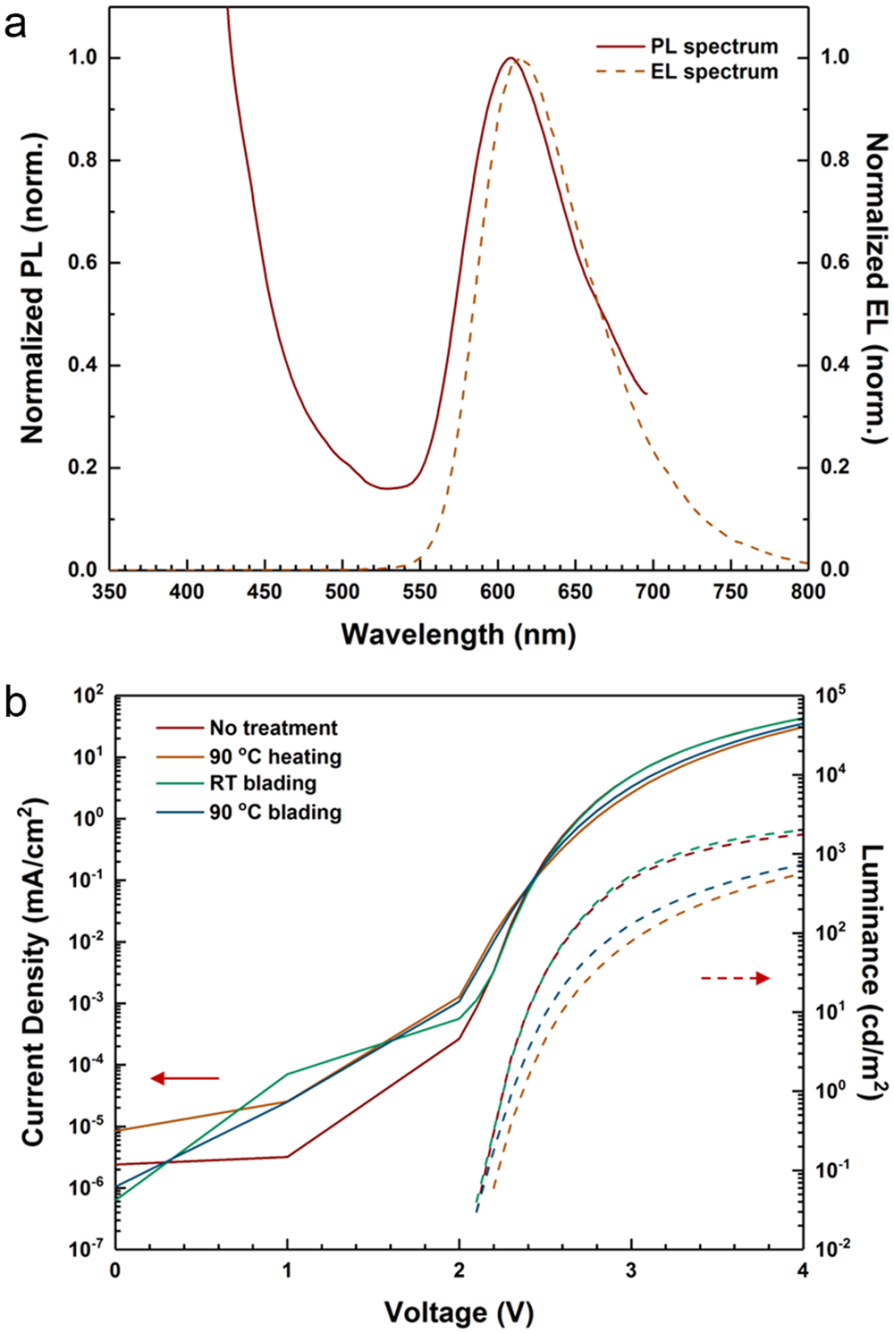
(**a**) Normalized PL spectrum of a neat C8-BTBT film doped with 10 wt% Ir(MDQ)_2_(acac) and normalized EL spectrum of a C8-BTBT OLED. (**b**) Current density and luminance vs. anode voltage characteristics of OLEDs fabricated with different treatment protocols.

The alignment and anisotropy of the C8-BTBT films make these layers highly interesting as matrix materials for OLED emission layers. Here, as a testbed, the red phosphorescent emitter, bis(2-methyldibenzo-[*f,h*]quinoxaline)(acetylacetonate)iridium(III) (Ir(MDQ)_2_(acac)), is doped into the C8-BTBT matrix. The phosphorescent emitter molecules, made of organometallic complexes comprising a heavy-metal atom, harvest energy from both singlet and triplet states, and are thus more likely to realize a highly efficient device compared to the fluorescent emitter molecules. Figure 6a shows the normalized PL spectrum (λ_exc_ = 370 nm) of a neat C8-BTBT film doped with 10 wt% Ir(MDQ)_2_(acac) and the normalized electroluminescence (EL) spectrum of a doped C8-BTBT-based OLED. Interestingly, the PL emission from C8-BTBT at λ < 500 nm is not fully suppressed in the mixture, even though the PL emission spectrum of C8-BTBT (see Fig. 3b) matches the peaks of Ir(MDQ)_2_(acac) absorbance well^54^. However, in the EL spectrum, only emission of Ir(MDQ)_2_(acac) is visible, indicating that excitons are predominantly generated on Ir(MDQ)_2_(acac) molecules directly, i.e. that the phosphorescent dopants provide another transport path for either electrons or holes in addition to transport across the matrix material and that excitons can be directly formed on the dopants (See the Supplementary Information for detailed discussion). The EL emission peak slightly shifts to higher wavelengths compared to the PL spectrum, which can be explained by a non-optimized OLED cavity.

Figure 6b compares the current density-luminance vs. anode voltage (or *J-L-V*) characteristics of OLEDs fabricated at different conditions, i.e. without and with the blading process. Interestingly, it is found that all OLEDs exhibit a similar current density even though the current is slightly decreased after thermal treatment at 90 °C, which is not in agreement with the increase in current density observed in pure C8-BTBT films (Fig. 5). Again, this can be explained by hopping of charge across the phosphorescent dopants in the emission layer. As depicted in Fig. S3a, if electrons encounter a large energy barrier at the emission layer/hole blocking layer interface, they are likely to be directly injected to dopants rather than the host matrix material^55^. In our case, the current density of OLEDs consisting of C8-BTBT doped with Ir(MDQ)_2_(acac) is higher than that of undoped C8-BTBT OLEDs as shown in Fig. S3b. This indicates the dopants play a significant role in charge transport, which leads an opposite behavior compared to the single charge carrier devices.

**Figure 7:**
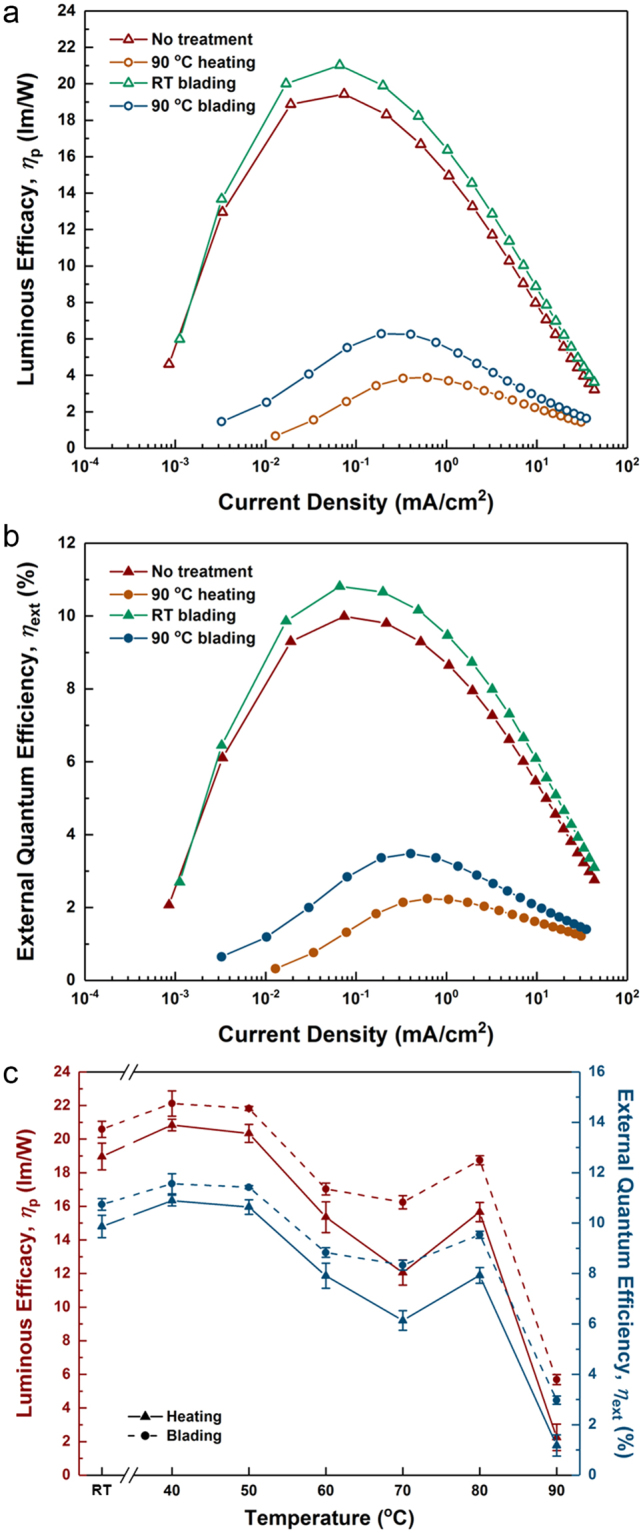
(**a**) Luminous efficacy (*η*_p_) and (**b**) EQE (*η*_ext_) vs. current density of OLEDs fabricated with different treatment protocols. (**c**) Comparison of luminous efficacy and EQE at *J* = 0.1 mA/cm^2^. The lines are drawn to guide the eye. The triangles and circles represent the samples treated by heating only and blading and heating, respectively. The data are averaged over 4 different OLED samples for each condition.

Figure 7a,b show the luminous efficacy (*η*_p_) and the external quantum efficiency (EQE) (*η*_ext_) vs. current density of our OLEDs fabricated under varying treatment conditions. It is clearly seen that both luminous efficacy and EQE are increased by the blading process compared to those of OLEDs treated only by heating. In Fig. 7c, the luminous efficacy and the EQE measured at *J* = 0.1 mA/cm^2^ are plotted for the different processing temperatures to allow for a direct comparison. Both quantities decrease gradually as the heating temperature increases up to 70 °C, but slightly increase at 80 °C. This increase can be explained by an improved charge balance in the emission layer resulting in a higher efficiency due to a molecular reorientation in C8-BTBT. However, a possible molecular segregation at 90 °C would hinder efficient intermolecular charge transport and exciton generation, leading to a decrease of the current density, the charge recombination rate, and overall efficiency. The PL quantum yield (PLQY) of doped C8-BTBT films is decreased after annealing at 90 °C compared to non-treated films, which also indicates that molecules partially segregate, resulting in less efficient energy transfer. In addition, relatively low PLQY values confirm the direct excitons formation on the phosphorescent dopants in C8-BTBT OLEDs (See Table S3 for a summary of PLQY values).

Using the blading process, the efficiency is significantly increased compared to only heating (more than two-fold at 90 °C). It is surprising that the blading process leads to an increase in efficiency even at low temperatures, which is shown to have no effect on charge transport inside the C8-BTBT layer. However, in contrast to the p-i-p and n-i-n devices shown in Fig. 5c, exciton formation takes place in only a few molecular layers on the top of the C8-BTBT layer, which might still be influenced by the blading process, even at RT (see also Fig. 4c in which the refractive indices are barely altered by the blading process at RT). In Fig. S4, the EL spectra measured with a linear polarizer parallel with and perpendicular to the blading direction are compared. Even though the optical properties of C8-BTBT layers can be altered by annealing and blading as seen in Fig. 4c, it is found that the optical behavior of emitted light in OLEDs in terms of polarization is not significantly influenced.

The OLEDs shown in Fig. 7b have a very early onset of the efficiency roll-off (less than 10 cd/m^2^), which is normally observed at much higher brightness^9,56^. The efficiency roll-off is usually rationalized by triplet-triplet annihilation (TTA), which, however, does not agree well with our experimental data as shown in Fig. S4a. On the other hand, as we describe above, there is a large charge carrier imbalance in the films due to the large electron injection barrier at the emission layer/hole blocking layer interface, resulting in a high charge carrier density and a sharp recombination zone. Thus, the strong efficiency roll-off seen in Fig. 7b is most likely not driven by TTA, but due to triplet-polaron quenching (See the Supplementary Information for detailed discussion). A relatively quick degradation of C8-BTBT OLEDs shown in Fig. S6 might be also attributed to exciton-polaron quenching led by a large amount of charge accumulation at the interface between the emission layer and the hole blocking layer as well as preferential charge carrier transport through particular paths. We expect that the design of novel liquid-crystalline semiconductor molecules with reduced LUMO levels ensuring a smaller injection barrier and better-balanced charge transport will reduce the efficiency roll-off behavior and improve the long-term device stability.

## Conclusion

A novel blading process capable of influencing the molecular structure of vacuum processed thin organic films is presented. By cross-polarized optical microscopy and spectroscopic ellipsometry it is shown that the molecular alignment in thin films of the semiconductor C8-BTBT is altered by annealing and the thermal-assisted blading, which consequently leads to altered optical properties. This change in alignment of the liquid-crystalline semiconductor leads to a profound improvement in hole and electron transport. It is shown that a Gaussian disorder model is in good agreement with the experimental results and can be used to quantitatively extract the mobility of single charge devices. Finally, p-i-n type OLEDs employing a liquid-crystalline emission layer are realized and it is shown that the light emission characteristics and efficiency are significantly improved by the blading process in dependence of the processing temperature conditions.

The results presented here open a new design space to optimize charge transport and to tune light propagation in OLEDs, which has the potential to lead to higher efficiencies. To take full advantage of this concept, new liquid-crystalline semiconductors have to be found that ensure a better charge balance. Furthermore, on a longer timeframe, designing liquid-crystalline emitter materials holds the promise to control the alignment of the emitting dipole of the molecule. Considering that a planar alignment of the emitter molecule has the potential to significantly reduce coupling to plasmonic losses in OLEDs, the orientation of the emitter dipole either of liquid-crystalline emitter molecule or inside the liquid-crystalline matrix in next experiments has to be studied thoroughly.

## Methods

### Materials

The acronyms of the materials used in this work are as follows. Spiro-TTB: 2,2′,7,7′-tetrakis-(N,N-di-methylphenylamino)-9,9-spiro-bifluorene, F_6_-TCNNQ: 2,2′-(perfluoronaphthalene-2,6-diylidene)dimalononitrile, NPB: N,N′-Bis(naphthalen-1-yl)-N,N′-bis(phenyl)-benzidine, C8-BTBT: 2,7-dioctyl[1]benzothieno[3,2-b][1]benzothiophene, Ir(MDQ)_2_(acac): bis(2-methyldibenzo-[*f,h*]quinoxaline)(acetylacetonate)iridium(III), BAlq: bis(2-methyl-8-quinolinolate)-4-(phenylphenolato)aluminium, NBphen: 2,9-bis(naphthalen-2-yl)-4,7-diphenyl-1,10-phenanthroline, Bphen: 4,7-diphenyl-1,10-phenanthroline, W_2_(hpp)_4_: tetrakis(1,3,4,6,7,8-hexahydro-2H-pyrimido[1,2*-a*]pyrimidinato)ditungsten(II), and HMDS: Hexamethyldisilazane.

### Thin-film characterization and Blading process

For the absorption and PL measurements of C8-BTBT only and C8-BTBT doped with 10 wt% Ir(MDQ)_2_(acac) films, 40 nm thick films are deposited on quartz substrates by thermal evaporation. The C8-BTBT films (60 nm) used for the cross-polarized optical microscopy and spectroscopic ellipsometry are prepared on silicon substrates covered with a thick silicon dioxide layer (300 nm). The blading process is carried out in a nitrogen-filled glovebox. A moving stage controlled by a stepping motor moves the sample substrate at the constant speed of 0.6 mm/s, translating the substrate from a hot plate to a supporting plate through an HMDS-treated silicon blade. While the substrate slides underneath the blade, the blading process is mainly determined by the weight of the blade (2.05 g) fixed at the angle of 12° with respect to the substrate. The speed of the stepping motor is controlled by an Arduino Uno board. The annealing process is performed for 1 min and the thermal-assisted blading process is controlled to ensure 1 min heating as well. The PL spectra, absorbance, and the optical constants of neat films are measured using a spectrofluorometer (Horiba Jobin Yvon-spex-Fluorog-3), a UV-Visible spectrophotometer (Agilent/HP 8453), and a spectroscopic ellipsometer (Horiba UVISEL), respectively. For the spectroscopic ellipsometry measurements, the polar angle (*θ*) is kept to be 70°. In particular, the modeling implemented in DeltaPsi2 software platform is used to calculate the optical constants from the measured spectroscopic ellipsometry data. The PLQY values of undoped and emitter-doped C8-BTBT films are measured using a spectrometer coupled to an integrating sphere with excitation at 325 nm (C9920-02 system, Hamamatsu).

### Device fabrication

The layers for the single charge devices are deposited on top of the glass substrates as follows; for n-i-n devices, C8-BTBT (varied thickness from 30 nm to 90 nm) is sandwiched between n-doped layers, i.e. NBphen (60 nm) doped with cesium carbonate (Cs_2_CO_3_) at 20 wt% or doped with W_2_(hpp)_4_ at 10 wt% and Bphen (60 nm) doped with Cs_2_CO_3_ at 20 wt%, and C8-BTBT is positioned between p-doped layers, Spiro-TTB (60 nm) doped with F_6_-TCNNQ at 4 wt%, in the case of p-i-p devices. Al (50 nm) electrodes are used as top and bottom contacts. The heating and blading processes are performed in a glovebox after the intrinsic layer, i.e. the C8-BTBT film, is deposited on the first doped layer, and subsequently the other layers are deposited. For the red OLEDs, the following layers are deposited on the indium-tin-oxide (ITO)-structured glass substrates from bottom to top: Spiro-TTB (60 nm) doped with F_6_-TCNNQ at 4 wt% as a hole transport layer, (HTL)/NPB (10 nm) as an electron blocking layer, (EBL)/C8-BTBT doped with 10 wt% Ir(MDQ)_2_(acac) as an emission layer, (EML) (30 nm)/BAlq (10 nm) as a hole blocking layer, (HBL)/Bphen doped with Cs_2_CO_3_ at 20 wt% as an electron transport layer, (ETL)/Al (100 nm) as top electrode. For OLEDs as well, the heating and blading processes are performed in a glovebox after the emission layer is deposited.

W_2_(hpp)_4_ is synthesized according to the procedure described in our previous report^57^ and the other organic materials are purchased from Lumtec Corp. All materials are used without further purification. Glass substrates are cleaned in an ultrasonic bath with acetone, methanol, and iso-propanol. Thermal evaporation is carried out in a vacuum chamber (Angstrom Engineering Inc.) at a base pressure of 0.5 ~ 1 × 10^−7^ Torr. The single charge carrier devices and OLEDs are structured by shadow masks defining an effective area of 2 mm × 2 mm and 4 mm × 4 mm, respectively. For the doped layers, the precise doping concentration is controlled by the evaporation rates of two materials, i.e. host matrix and guest dopant. Each deposition rate is individually monitored by two quartz crystal microbalances.

### Device characterization

The electrical and optical characteristics of our devices are measured using a semiconductor parameter analyzer (4200-SCS, Keithley) and a photodiode (FDS1010, Thorlab) in a nitrogen-filled glovebox under dark ambient condition. Luminance, EQE and luminous efficiencies are carefully calibrated by data measured using an integrating sphere (FOIS-1, OceanOptics). The EL spectra of the Ir(MDQ)_2_(acac) emission are obtained using a spectrometer (USB4000, OceanOptics) coupled to an optical fiber (OceanOptics). The lifetime of OLEDs is measured by monitoring the relative luminance over time using a photodiode (PDA100A-EC, Thorlab) under a constant bias stress.

### Data Availability

All data supporting this study are included in this published article and Supplementary Information file. Additional information and the datasets are available from the corresponding author on reasonable request.

## Electronic supplementary material

Supplementary Information
