# Supplementary material for: Tuning charge carrier transport and optical birefringence in liquid-crystalline thin films: A new design space for organic light-emitting diodes
Source: Sci Rep. 2018 Jan 15;8:699. doi: 10.1038/s41598-018-19157-9 (PMC5768873; doi:10.1038/s41598-018-19157-9)
Supplement: Supplementary file 1 — Supplementary Information [file 41598_2018_19157_MOESM1_ESM.pdf]

**Supplementary Information**

# Tuning charge carrier transport and optical birefringence in liquid-crystalline thin films: A new design space for organic light-emitting diodes

Chang-Min Keum, Shiyi Liu, Akram Al-Shadeedi, Vikash Kaphle, Michiel Koen Callens, Lu Han, Kristiaan Neyts, Hongping Zhao, Malte C. Gather, Scott D. Bunge, Robert Twieg, Antal Jakli, and Björn Lüssem

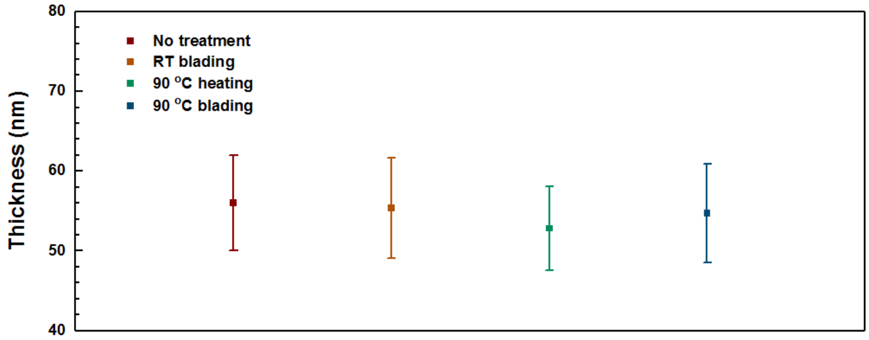


**Figure S1.** The film thicknesses determined by spectroscopic ellipsometry show no substantial change after heating and blading at room temperature and 90 °C. The error bars represent the results of the software modelling fitting to the measured ellipsometry data.

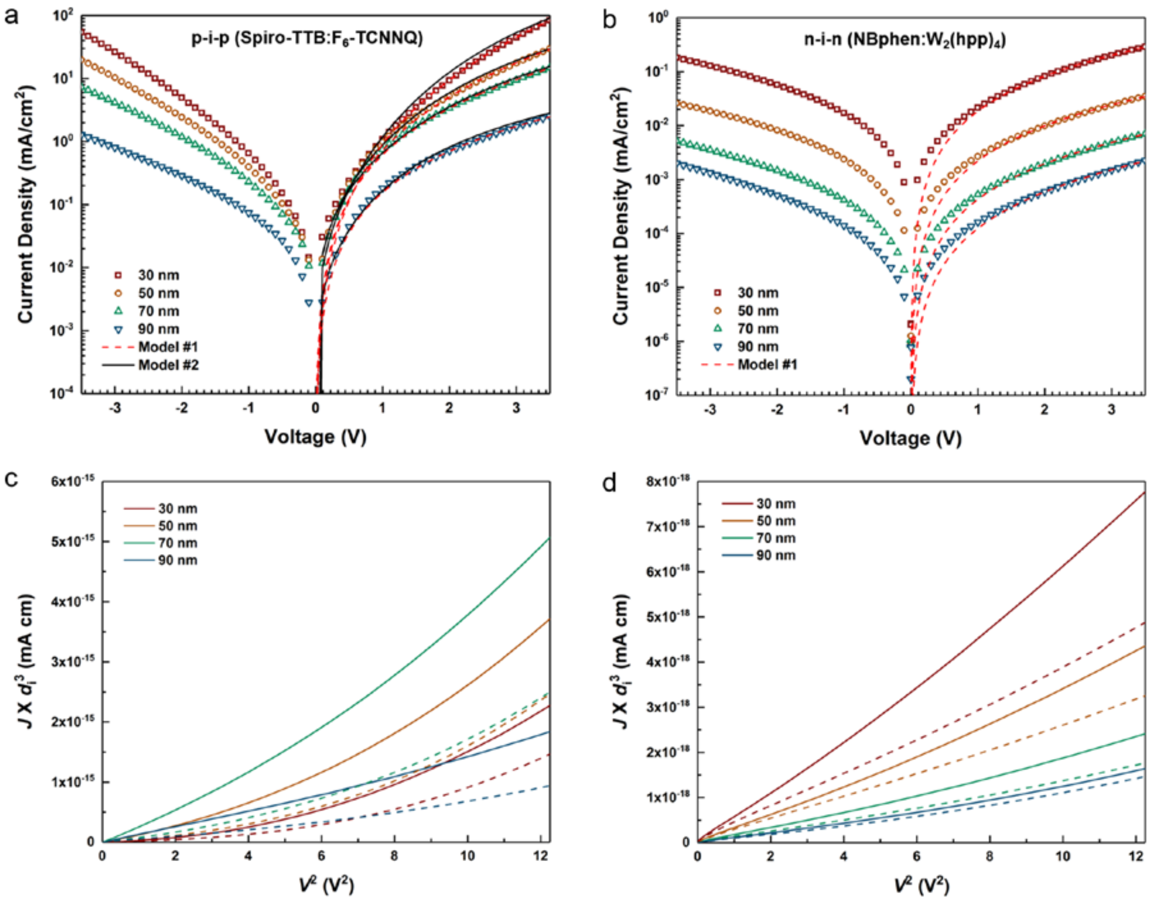

**Figure S2.** Current density of **(a)** p-i-p (Spiro-TTB doped with $F_6$-TCNNQ) and **(b)** n-i-n (NBphen doped with $W_2(hpp)_4$) devices with varied thickness of the intrinsic layer (C8-BTBT) and comparison between experimental data and simulated results. The red dashed and black solid lines represent fitting results using the modified M-G model (Model #1) and EGDM (Model #2), respectively. **(c), (d)** $J \times d_i^3$ vs. $V^2$ relations replotted from a), b), respectively. The solid and dashed lines represent the current measured at positive or negative voltages, respectively (The voltage is applied to the top electrode while the bottom electrode is grounded).

In Figs. S2a and S2b, current density vs. voltage characteristics of as-prepared p-i-p and n-i-n devices with varied thickness of the intrinsic layer are plotted. It is found that the current density systematically scales with the thickness of the intrinsic layer for both p-i-p and n-i-n devices. To analyze the current density–voltage characteristics of single charge carrier devices, the Mott-Gurney (M-G) relation given by Eq. S1 is widely used[1].

$$J = \frac{9}{8}\varepsilon_r\varepsilon_0\mu\frac{V^2}{d_i^3} \qquad \text{(S1)}$$

where $\varepsilon_r$ is the dielectric constant of the intrinsic layer, $\varepsilon_0$ permittivity, $\mu$ the mobility, $V$ applied voltage, and $d_i$ the intrinsic layer thickness. In Figs. S2c and S2d, $J \times d_i^3$ of single charge carrier devices shown in Figs. S2a and S2b, respectively, is plotted as a function of $V^2$. Ideally, if Eq. S1 is valid for the experimental data, the slope in Figs. S2c and S2d should be linear and identical for every intrinsic layer thickness. However, it is clearly seen this model is not in agreement with our experimental results. The discrepancy between the M-G model and the experimental results occurs mostly because this model is only satisfactory under the strict assumptions that i) the charge carrier mobility is constant and in particular independent of the electric field and the charge carrier density and ii) that any trapping effects are absent[2].

The Poole-Frenkel mobility model given by $\mu = \mu_0 \exp(\gamma\sqrt{V/d_i})$ effectively describes a field dependency which allows a better fit than M-G model for the charge transport modelling[3]. As summarized in Table 1, the modified M-G model provides relatively reliable fitting results, i.e. fitting parameters are in the same order for different intrinsic layer thickness, yet it is valid in only a limited field range as seen in Fig. 5 as well as Figs. S2a and S2b.

**Table S1.** Summary of fitting parameters ($\mu_0$ zero-field mobility and $\gamma$ the characteristic factor) obtained from Figs. S2a and S2b by the modified M-G model (Model #1) given by Eq. 2.

| Devices | p-i-p | | n-i-n | |
|---|---|---|---|---|
| $d_i$ (nm) | $\mu_0$ (cm$^2$/Vs) | $\gamma$ ((cm/V)$^{1/2}$) | $\mu_0$ (cm$^2$/Vs) | $\gamma$ ((cm/V)$^{1/2}$) |
| 30 | $7.87 \times 10^{-9}$ | $4.55 \times 10^{-3}$ | $1.29 \times 10^{-9}$ | $5.09 \times 10^{-4}$ |
| 50 | $7.68 \times 10^{-8}$ | $3.45 \times 10^{-3}$ | $7.05 \times 10^{-10}$ | $6.86 \times 10^{-4}$ |
| 70 | $3.31 \times 10^{-7}$ | $2.25 \times 10^{-3}$ | $3.59 \times 10^{-10}$ | $9.42 \times 10^{-4}$ |
| 90 | $2.82 \times 10^{-7}$ | $1.00 \times 10^{-3}$ | $2.00 \times 10^{-10}$ | $1.42 \times 10^{-3}$ |

The extended Gaussian disorder model (EGDM) has been developed to take the charge carrier density dependency of the mobility into account[1]. This model is based on a 3D master equation, which represents charge carrier hopping between sites on a cubic lattice in disordered organic materials assuming a Gaussian distribution with site energies:

$$N(E) = \frac{N_t}{\sqrt{2\pi\sigma^2}} \exp\left(-\frac{E^2}{2\sigma^2}\right), \quad \text{(S2)}$$

where the density of hoping sites $N_t = 1/a^3$ with a lattice spacing $a$ and the width $\sigma$. It was shown that the 3D simulation modelling can be expressed as 1D scalar EGDM mobility as a function of temperature ($T$), charge carrier density ($p$), and electric field ($E$)[4,5]:

$$\mu(T,p,E) = \mu(T,p) f(T,E) \quad \text{(S3a)}$$

where

$$\mu(T,p) = \mu_0(T) \exp\left[\frac{1}{2}(\hat{\sigma}^2 - \hat{\sigma})(2pa^3)^{\delta}\right], \quad \text{(S3b)}$$

$$\mu_0(T) = \mu_0^* \exp(-C\hat{\sigma}^2), \quad \text{(S3c)}$$

$$\delta = 2[\ln(\hat{\sigma}^2 - \hat{\sigma}) - \ln(\ln 4)]/\hat{\sigma}^2, \quad \text{(S3d)}$$

$$f(T,E) = \exp\left[0.44\left(\hat{\sigma}^{\frac{3}{2}} - 2.2\right)\left(\sqrt{1 + 0.8\hat{E}^2} - 1\right)\right]. \quad \text{(S3e)}$$

Here, $\mu_0^*$ denotes the mobility at the zero-field and zero-density condition. The dimensionless disorder parameter $\hat{\sigma} = \sigma/k_B T$ and the dimensionless electric field $\hat{E} = Eea/\sigma$ are used in Eq S3. The simulation parameters used for our p-i-p devices are summarized in Table S2.

**Table S2.** Summary of simulation parameters used in the EGDM numerical analysis of hole transport in p-i-p devices shown in Fig. 5c and Fig. S2a.

| $N_t$ (cm$^{-3}$) | $C$ | $\Delta$HOMO* (eV) | $\sigma$ (eV) | $\mu_0$*(cm$^2$/Vs) | Ionized dopants (cm$^{-3}$) |
|---|---|---|---|---|---|
| $10^{20}$ | 0.42 | 0.19 | 0.136 ± 0.001 | $(2.2 \pm 1.2) \times 10^{-7}$ | $(3.5 \pm 1.5) \times 10^{17}$ |

* $\Delta$HOMO = HOMO of intrinsic layer – HOMO of p-doped layer

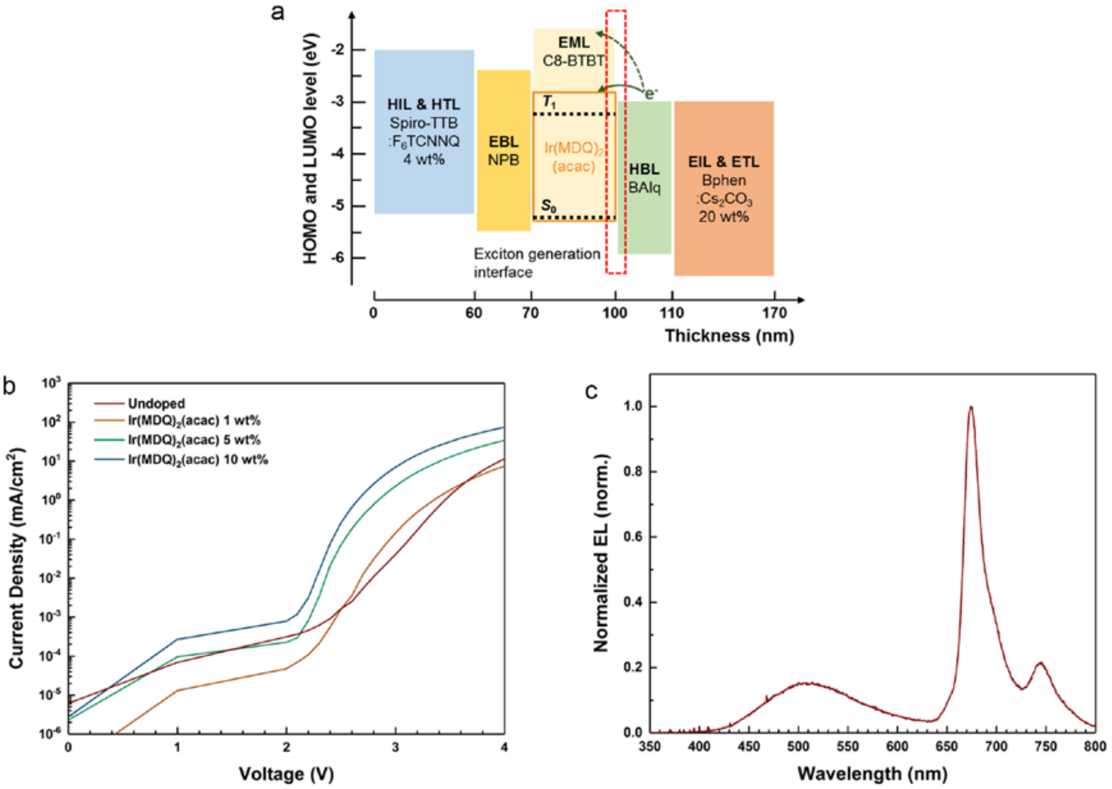


**Figure S3. (a)** Energy level diagram of C8-BTBT OLEDs. In the emission layer, the orange box line depicts the energy level of Ir(MDQ)$_2$(acac) and the black dotted lines represent the exciton states corresponding to the light emission. **(b)** *J-V* characteristics of OLEDs comprised of undoped C8-BTBT and C8-BTBT doped with 1, 5, and 10 wt% Ir(MDQ)$_2$(acac) as the emission layer **(c)** EL spectrum of a dopant-free C8-BTBT OLED device.

Figure S3a schematically depicts an energy level diagram of C8-BTBT OLEDs studied in this work, where the corresponding highest occupied molecular orbital (HOMO) and lowest unoccupied molecular orbital (LUMO) levels are visualized with the thickness of each layer. Figure S3b shows the current density vs. voltage curves of OLEDs containing undoped C8-BTBT and C8-BTBT doped with 1, 5, and 10 wt% Ir(MDQ)$_2$(acac) as the emission layer. The current density of C8-BTBT OLEDs systematically increases as the doping concentration increases, which indicates that the phosphorescent dopants enhance the

hopping transport in the emission layer. In case of 1 wt% doping, the current density is slightly lower than undoped OLEDs at a high voltage. This might be because the dopants are disturb the molecular arrangement of C8-BTBT, which hinders efficient charge transport, even though they generate a certain amount of additional charge transport paths. Figure S3c shows the EL spectrum of an undoped C8-BTBT OLED. Interestingly, the peak position and shape of the EL spectrum are completely different from the PL spectra of C8-BTBT thin-films shown in Fig. 3b. This might be attributed to exciplex formation, i.e. the formation of bimolecular excited states[6,7], at the interface between BAlq and C8-BTBT layers due to a large injection barrier. The exciplex formation would be an indication as well that electron injection into the C8-BTBT layer is not efficient, which is consistent with the n-i-n devices electrical characteristics and *J-V* characteristics in Fig. S3b. On the other hand, the OLEDs made with the emission layer of C8-BTBT doped with $Ir(MDQ)_2(acac)$ show the exactly identical EL spectra with PL of such films as seen in Fig. 6a, indicating that excitons are predominantly generated on $Ir(MDQ)_2(acac)$ molecules directly for electrical excitation.

**Table S3.** Summary of photoluminescence quantum yield (PLQY) ($\Phi_{PL}$) of undoped and $Ir(MDQ)_2(acac)$-doped C8-BTBT films (thickness, 100 nm) treated with varied conditions.

| PLQY ($\Phi_{PL}$) | Undoped C8-BTBT | C8-BTBT: $Ir(MDQ)_2(acac)$ 10 wt% |
|---|---|---|
| No treatment | 0.035 | 0.059 |
| 90 °C heating | 0.033 | 0.041 |
| RT blading | 0.035 | 0.060 |
| 90 °C blading | 0.039 | 0.044 |

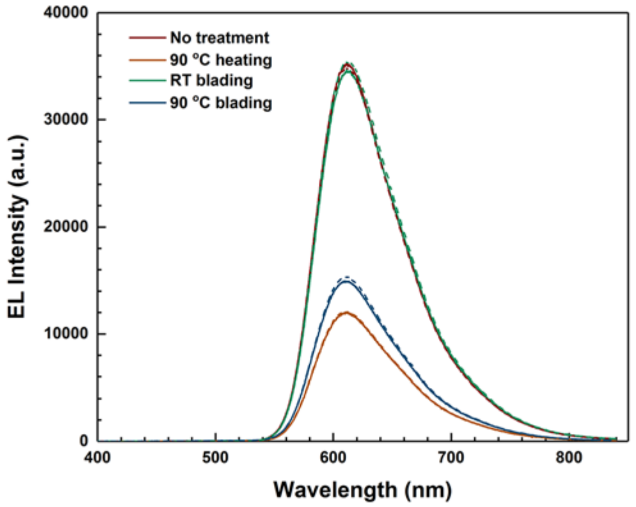


**Figure S4.** Comparison of EL spectra measured with a linear polarizer parallel with (solid lines) and perpendicular to (dashed lines) the blading direction

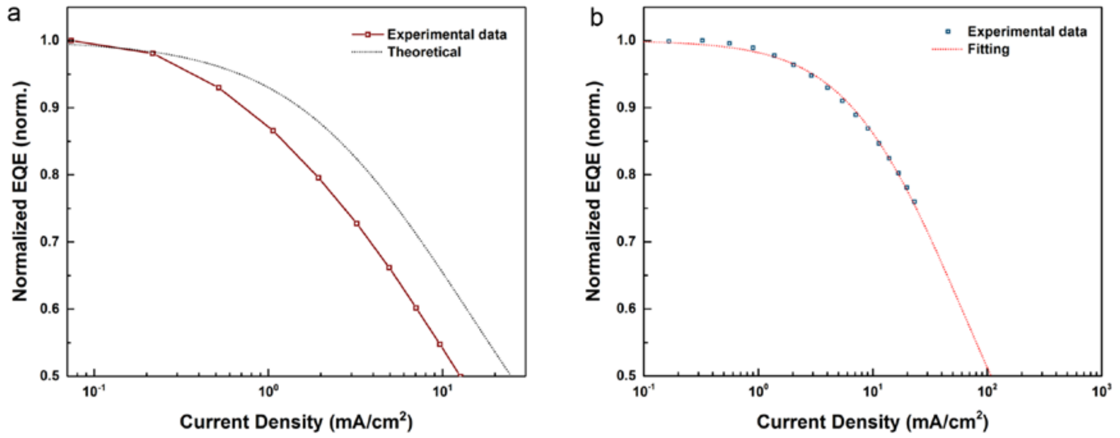


**Figure S5.** Normalized EQE vs. current density of **(a)** C8-BTBT OLED and **b)** NPB OLED. The current density at which the EQE is half of $EQE_0$ is defined as $J_0$. The black and red dotted line in a) and **(b)** denote a theoretical result and a fitting curve, respectively, obtained by Eq. (S5a).

In Fig. S4, the normalized EQE vs. current density of C8-BTBT OLED and NPB OLED are plotted. In NPB OLED, NPB is employed as the host matrix in the emission layer instead of C8-BTBT. The current density at which the EQE is half of $EQE_0$ is defined as $J_0$. Here, $EQE_0$ represents technically the maximum EQE, which is commonly defined as the efficiency in the absence of triplet-triplet annihilation (TTA). Based on assumptions that TTA is the only relevant exciton quenching process and TTA occurs only in excited states of guest, the rate of the triplet exciton generation in OLEDs regarding the electrical charge supply is described by the following equation[8,9]:

$$\frac{d}{dt}n(t) = -\frac{n(t)}{\tau^*} - \frac{1}{2}k_{\mathrm{TT}}n(t)^2 + \frac{J}{qw} \qquad \text{(S4)}$$

Here, $n$(t) is the triplet exciton density, $\tau^*$ is the effective emitter lifetime, $k_{\mathrm{TT}}$ is the TTA rate constant, $q$ is the elementary charge, and $w$ is the width of the exciton formation zone. The normalized EQE can be solved as following from the steady-state solution of Eq. S4 together with $J_0$ and $EQE_0$ defined above.

$$\frac{\mathrm{EQE}}{\mathrm{EQE}_0} = \frac{J_0}{4J}\left(\sqrt{1+\frac{8J}{J_0}} - 1\right) \qquad \text{(S5a)}$$

where

$$J_0 = \frac{4qw}{k_{\mathrm{TT}}\tau^{*2}} \qquad \text{(S5b)}$$

In Fig. S4a, the black dotted line denotes a theoretical result of the non-treated C8-BTBT OLED obtained by Eq. S5a by assuming that only data at a low current density (< 0.02 $mA/cm^2$ in our case) satisfy the TTA model. The experimental data show significant deviation from the theoretical model in contrast to the NPB OLED shown in Fig. S4b in

which the theoretical model is fitting well. This means that not only TTA but other exciton quenching mechanisms as well are involved in C8-BTBT OLEDs. This might originate from triplet annihilation due to triplet-polaron quenching within a sharp recombination zone at the EML/HBL interface (see Fig. S3a). Comparing $J_0$ values extracted for C8-BTBT OLED ($J_0$ = 24.8 mA/cm$^2$) and NPB OLED ($J_0$ = 100.2 mA/cm$^2$) from Figs. S4a and S4b, respectively, we can estimate the exciton formation zone of C8-BTBT OLED is about 4 times narrower than that of NPB OLED according to Eq. S5b, with an assumption that $\tau^*$and $k_{TT}$ of Ir(MDQ)$_2$(acac) would not be significantly varied independent of the host matrix. The probability of TTA should be higher in case of C8-BTBT within a narrow exciton formation zone, leading to early onset of the efficiency roll-off as well even though the brightness is not much high.

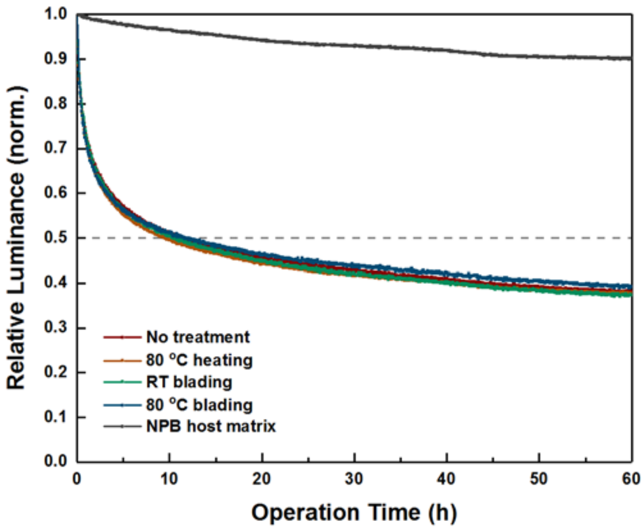


**Figure S6.** Lifetime of C8-BTBT OLEDs treated with varied annealing and blading conditions and NPB OLED as a reference. The relative luminance is measured every 5 min under a constant bias stress at $J$ = 1 mA/cm$^2$.

Figure S6 shows the degradation of C8-BTBT OLEDs and NPB OLED in terms of the luminance over time under a constant bias stress at $J$ = 1 mA/cm$^2$. The initial luminance is 110 ~ 130 cd/m$^2$ for C8-BTBT OLEDs and 300 cd/m$^2$ for NPB OLED. The C8-BTBT OLED devices non-treated, treated with annealing at 80 °C, blading at RT, and blading at 80 °C exhibit the lifetime of 11.0 h, 9.4 h, 10.3 h, and 11.6 h, respectively, for 50 % of the initial luminance and the lifetime of NPB OLED up to 90 % of initial luminance is 62.6 h.

## References


1 Pasveer, W. F. *et al.* Unified description of charge-carrier mobilities in disordered semiconducting polymers. *Phys. Rev. Lett.* **94**, 206601, (2005).

2 van Mensfoort, S. L. M. & Coehoorn, R. Effect of gaussian disorder on the voltage dependence of the current density in sandwich-type devices based on organic semiconductors. *Phys. Rev. B* **78**, (2008).

3 Murgatroyd, P. N. Theory of space-charge-limited current enhanced by frenkel effect. *J. Phys. D Appl. Phys.* **3**, 151, (1970).

4 Schober, M. *et al.* Quantitative description of charge-carrier transport in a white organic light-emitting diode. *Phys. Rev. B* **84**, 165326, (2011).

5 Coehoorn, R. & Bobbert, P. A. Effects of gaussian disorder on charge carrier transport and recombination in organic semiconductors. *Phys. Status Solidi A* **209**, 2354, (2012).

6 Kalinowski, J., Cocchi, M., Virgili, D., Fattori, V. & Williams, J. A. G. Mixing of excimer and exciplex emission: A new way to improve white light emitting organic electrophosphorescent diodes. *Adv. Mater.* **19**, 4000, (2007).

7 Goushi, K. & Adachi, C. Efficient organic light-emitting diodes through up-conversion from triplet to singlet excited states of exciplexes. *Appl. Phys. Lett.* **101**, 023306, (2012).

8 Baldo, M. A., Adachi, C. & Forrest, S. R. Transient analysis of organic electrophosphorescence. Ii. Transient analysis of triplet-triplet annihilation. *Phys. Rev. B* **62**, 10967, (2000).

9 Murawski, C., Liehm, P., Leo, K. & Gather, M. C. Influence of cavity thickness and emitter orientation on the efficiency roll-off of phosphorescent organic light-emitting diodes. *Adv. Funct. Mater.* **24**, 1117, (2014).
